# Factors Associated With Willingness to Use Pre-Exposure Prophylaxis in Brazil, Mexico, and Peru: Web-Based Survey Among Men Who Have Sex With Men

**DOI:** 10.2196/13771

**Published:** 2019-06-17

**Authors:** Thiago Silva Torres, Kelika A Konda, E Hamid Vega-Ramirez, Oliver A Elorreaga, Dulce Diaz-Sosa, Brenda Hoagland, Steven Diaz, Cristina Pimenta, Marcos Benedetti, Hugo Lopez-Gatell, Rebeca Robles-Garcia, Beatriz Grinsztejn, Carlos Caceres, Valdilea G Veloso, ImPrEP Study Group

**Affiliations:** 1 Instituto Nacional de Infectologia Evandro Chagas, Fundação Oswaldo Cruz (INI/Fiocruz) Rio de Janeiro Brazil; 2 Centro de Investigación Interdisciplinaria en Sexualidad Sida y Sociedad, UPCH Lima Peru; 3 Condesa & Condesa-Iztapalapa Specialized Clinics Mexico City Mexico; 4 National Institute of Psychiatry Ramon de la Fuente Muñiz Mexico City Mexico; 5 Center for Prevention and Comprehensive Healthcare for HIV/AIDS of Mexico City Mexico City Mexico; 6 Brazilian Ministry of Health Brasília Brazil; 7 National Institute of Public Health Mexico City Mexico

**Keywords:** pre-exposure prophylaxis, men who have sex with men, prevention, Latin America, surveys and questionnaires

## Abstract

**Background:**

HIV disproportionally affects key populations including men who have sex with men (MSM). HIV prevalence among MSM varies from 17% in Brazil and Mexico to 13% in Peru, whereas it is below 0.5% for the general population in each country. Pre-exposure prophylaxis (PrEP) with emtricitabine/tenofovir is being implemented in the context of combination HIV prevention. Reports on willingness to use PrEP among MSM have started to emerge over the last few years. Previously reported factors associated with willingness to use PrEP include awareness, higher sexual risk behavior, and previous sexually transmitted infection.

**Objective:**

This study aimed to evaluate the factors associated with willingness to use daily oral PrEP among MSM in 3 Latin American, middle-income countries (Brazil, Mexico, and Peru).

**Methods:**

This Web-based, cross-sectional survey was advertised in 2 gay social network apps (Grindr and Hornet) used by MSM to find sexual partners and on Facebook during 2 months in 2018. Inclusion criteria were being 18 years or older, cisgender men, and HIV-negative by self-report. Eligible individuals answered questions on demographics, behavior, and PrEP (awareness, willingness to use, barriers, and facilitators). Multivariable logistic regression modeling was performed to assess the factors associated with willingness to use daily oral PrEP in each country.

**Results:**

From a total sample of 43,687 individuals, 44.54% of MSM (19,457/43,687) were eligible and completed the Web-based survey—Brazil: 58.42% (11,367/19,457), Mexico: 30.50% (5934/19,457), and Peru: 11.08% (2156/19,457); median age was 28 years (interquartile range: 24-34), and almost half lived in large urban cities. Most participants were recruited on Grindr (69%, 13,349/19,457). Almost 20% (3862/19,352) had never tested for HIV, and condomless receptive anal sex was reported by 40% (7755/19,326) in the previous 6 months. Whereas 67.51% (13,110/19,376) would be eligible for PrEP, only 9.80% (1858/18,959) of participants had high HIV risk perception. PrEP awareness was reported by 64.92% (12,592/19,396); this was lower in Peru (46.60%, 1002/2156). Overall, willingness to use PrEP was reported by 64.23% (12,498/19,457); it was highest in Mexico (70%, 4158/5934) and lowest in Peru (58%, 1241/2156). In multivariate regression models adjusted for age, schooling, and income in each country, willingness to use PrEP was positively associated with PrEP awareness and PrEP facilitators (eg, free PrEP and HIV testing) and negatively associated with behavioral (eg, concerned by daily pill regimen) and belief barriers (eg, sexual partners may expect condomless sex).

**Conclusions:**

In this first cross-country, Web-based survey in Latin America, willingness to use PrEP was found to be high and directly related to PrEP awareness. Interventions to increase awareness and PrEP knowledge about safety and efficacy are crucial to increase PrEP demand. This study provides important information to support the implementation of PrEP in Brazil, Mexico, and Peru.

## Introduction

### Background

HIV continues to be a major health problem worldwide. The Latin American region has the fourth largest number of individuals living with HIV (1.8 million accumulated cases) and is fifth with regard to new HIV infections (96,000). About 90% of new HIV infections in 2016 in Latin America occurred in 10 countries, including Brazil (49%), Mexico (13%), and Peru (4%) [1]. In this region, HIV disproportionally affects key populations, and these are primarily gay, bisexual, and other men who have sex with men (MSM) and transgender women [2]. HIV prevalence among MSM varies from 17% in Brazil [3] and Mexico [4] to 13% in Peru [5], whereas it is below 0.5% for the general population in each country [6-8]. This continuing burden highlights the need for a more energetic, integrated, and strategic focus on combination HIV prevention among this population.

Pre-exposure prophylaxis (PrEP) with daily oral emtricitabine/tenofovir (FTC/TDF) is an effective biomedical prevention strategy to reduce HIV acquisition among MSM [9-12]. PrEP guidelines were first published in 2012 by the US Centers for Disease Control and Prevention [13], followed by the World Health Organization (WHO) in 2014 [14], and in 2017, by the Brazilian Ministry of Health (MoH) [15]. PrEP is being implemented by public health services in Brazil [16], and it is being considered for implementation in Mexico and Peru, even though neither country has issued guidance with regard to PrEP.

Reports on PrEP awareness, willingness to use, and acceptability among MSM have started to emerge over the last few years. Few MSM were aware of PrEP (<50%) in 11 studies in low- and middle-income countries [17]. Willingness to use PrEP among MSM varies by country and time, ranging from 32% to 92% [18], suggesting a diversity of factors associated with willingness to use PrEP. Previously reported factors associated with willingness to use PrEP included the following: awareness, low cost of PrEP, higher sexual risk, previous sexually transmitted infection (STI), and unwillingness to use condoms [19]. However, these factors may vary according to the characteristics of the region or country and may change rapidly. Recent reports of awareness and willingness to use PrEP among MSM are available for Brazil [20-22], but no information was captured after the availability of PrEP in the public health system. Conversely, no recent reports are available for either Mexico or Peru.

The Implementation PrEP Project (ImPrEP) is the first transnational project in Latin America aiming to generate evidence on the feasibility, acceptability, and cost-effectiveness of PrEP among key populations (MSM and transgender people) specific to the cultural contexts and health systems in Brazil, Mexico, and Peru. Within ImPrEP, several studies are being conducted, including a PrEP demonstration study with 7500 MSM and transgender people in these countries. The results of ImPrEP [23] will permit stakeholders to evaluate and incorporate PrEP as part of combination HIV prevention within their countries. The data presented herein are from a formative survey conducted for ImPrEP, with the aim to better understand the characteristics, sexual behavior, and the knowledge and opinions regarding PrEP among MSM.

### Objective

This study evaluated the factors associated with willingness to use PrEP among MSM in Brazil, Mexico, and Peru.

## Methods

### Study Design

This was a cross-sectional, Web-based survey for MSM from 3 countries in Latin America: Brazil, Mexico, and Peru. Individuals who met the eligibility criteria (age ≥18 years, cisgender men, and HIV uninfected by self-report) and provided informed consent were directed to the Web-based questionnaire.

The questionnaire was conducted using SurveyGizmo in Brazil and SurveyMonkey in Mexico and Peru. The project was advertised on 2 geosocial networking apps for sexual encounters among men (Hornet and Grindr) and Facebook social media. On Facebook, advertisements focused toward male gender, country (Brazil, Mexico, or Peru), and related interests, applying keywords frequently used by gay and bisexual population, for instance, gay pride, gay community, and homophobia. Hornet users received 2 inbox messages with a link to the survey, and Grindr users received pop-up advertisements, 1 per week for 2 months. The advertisements were launched in March for Brazil and Peru and in June for Mexico, all in 2018. The questionnaire remained open for 2 months in each country. The informed consent stated clearly that the survey was targeting MSM. Another survey targeting transgender people within ImPrEP is still ongoing. No incentives were provided for answering the survey.

### Variables

#### Sociodemographics

Age was categorized in the following 3 brackets: 18 to 24, 25 to 34, and ≥35 years. Race was dichotomized as white versus nonwhite (black, mixed-race, Asian, or indigenous) for Brazil and Peru; in Mexico, this question was dichotomized as indigenous versus nonindigenous. Definition of monthly income varied in each country (see Table 1 footnote). Schooling was dichotomized by those who had less than or complete secondary education versus any postsecondary education.

#### HIV Testing and HIV Risk Perception in the Next Year

Individuals were asked when they were last tested for HIV. The options were as follows: last 3 months, last 6 months, last year, more than 1 year, and never tested. We dichotomized this question in never versus at least once in a lifetime for the multivariate analyses. HIV risk perception was assessed with the question *What is your chance of getting HIV in the next year?* with possible options grouped into 3 categories for analysis: low (none/low risk), middle (some risk), and high (high risk/certainty of infection) [22].

#### Sexual Behavior and Preliminary Eligibility for Pre-Exposure Prophylaxis (Adapted From the World Health Organization Risk Criteria for Pre-Exposure Prophylaxis)

Sexual behavior was assessed in the last 6 months with the following: number of male sexual partners (0, 1-5, 6-10, and ≥10); condomless receptive anal sex, condomless insertive anal sex, sex with HIV-positive male partner, sex with a male partner of unknown HIV status, sex under the influence of alcohol, and chemsex or sex under the influence of excitatory drugs (all dichotomized yes/no).

We evaluated preliminary eligibility for PrEP considering the ImPrEP demonstration study’s risk-related inclusion criteria, which were adapted from the WHO recommendations for PrEP use (all in the past 6 months): reporting unprotected sex, having an HIV-positive male partner, exchanging sex for money, or having an STI [24]. Individuals were then dichotomized (yes/no) as preliminarily eligible for PrEP based on risk if they fulfilled any of the listed criteria.

#### Substance Use

Binge drinking [25] was evaluated as follows: *In the last six months, did you drink five or more drinks in a couple of hours?* A dose was defined as 1 can of beer (300 mL) or 1 glass of wine (120 mL) or 1 shot of distilled alcohol (30 mL of ex. cachaça, vodka, whisky, tequila, mezcal, or pisco). Substance use in the past 6 months included the following: tobacco, marijuana, hash, stimulants (cocaine, crack, amphetamines, and 4-hydroxybutanoic acid), hallucinogens (lysergic acid diethylamide, ketamine), *poppers* (alkyl nitrites), and erectile dysfunction drugs.

#### Awareness, Willingness to Use, Barriers, and Facilitators of Daily Oral Pre-Exposure Prophylaxis

Awareness was assessed as follows: *Have you ever heard of PrEP?* and dichotomized yes/no. A brief explanation of PrEP was provided after respondents had answered the PrEP awareness question. PrEP use was categorized as follows: *never*, *current*, and *past*. We asked respondents to rank 3 PrEP regimens from 1-most preferred to 3-least preferred: daily oral (1 FTC/TDF pill/day), event-driven (2 pills before intercourse and 1 pill 24 hours and 48 hours after), and injectable (injection every 2 months).

Willingness is understood as an intentional behavior based on 2 principles: (1) recognition of the behavioral objective (ie, taking PrEP) and (2) strategies implemented to achieve this objective. The second principle can be *experiential processes*, which refer to cognitive strategies (eg, believing that PrEP will protect against HIV) or *behavioral processes*, which are strategies to produce and maintain the behavior (eg, setting an alarm as a reminder to take PrEP) [26]. We used a 5-point Likert scale to assess willingness to use PrEP, anticipated risk compensation, and if participants would use PrEP provided by the MoH at no cost. Willingness to use PrEP was defined as responding *highly likely* to the statement *I would use one daily pill for PrEP*. Anticipated risk compensation was defined as responding *highly likely* or *likely* to the following statement: *I would not use a condom if I used PrEP*. PrEP if provided by the MoH was defined as responding *highly likely* to the statement *I would use PrEP if part of a MoH program*. The inclusion of the *likely* category only for anticipated risk compensation was to be more conservative with this as a potentially negative outcome of PrEP use.

Barriers and facilitators to daily oral PrEP were accessed using a 5-point Likert scale (*very important* to *not important*) [27]. For descriptive statistics, barriers were defined as responding *very important* or *important*, and facilitators were defined as responding *very important*. The inclusion of *important* category only for barriers was to more broadly identify relevant obstacles of willingness to use PrEP. Barriers were grouped into the following: informational (eg, concerned by short-term or long-term side effects, that antiretroviral therapy would not work if they became HIV infected while on PrEP, and by lack of 100% protection against HIV), behavioral (eg, concerned by daily pill regimen, talking to a doctor about sex, and having quarterly HIV/STI testing), and beliefs (eg, concerned that taking PrEP implies being at risk of HIV acquisition, sexual partners may expect condomless anal sex, people may assume they are HIV-positive, and people may ask why they are taking pills). For facilitators, we assessed the following: free PrEP, access to free HIV testing, access to other free exams (eg, HIV/STI testing), access to personal PrEP counseling, adherence support from apps, support, and counseling about sex life. Using these groupings, we performed confirmatory factor analysis and calculated the Cronbach alpha to verify if these items could be grouped (each Cronbach alpha was >.70).

Percentage of willingness to use daily oral prep is also given per region. For Brazil, region was categorized according to geopolitical regions: North (7 states), Northeast (9 states), Central-West (3 states and Federal District), South (3 states), and Southeast (2 states). São Paulo and Rio de Janeiro are part of Southeast Brazil, but they were maintained separated in the analysis because of the high number of responders from these 2 states. For Peru, regions are grouped according to their geographical characteristics and political division: Lima (Lima city and Callao), Coast (Lima region and other coastal cities), Sierra (cities of the northern, central, and southern highlands), and Jungle. For Mexico, the regions were Northwest (Baja California, Baja California Sur, Chihuahua, Durango, Sinaloa, and Sonora), Northeast (Coahuila, Nuevo León, and Tamaulipas), West (Nayarit, Jalisco, Colima, and Michoacán), East (Puebla, Veracruz, Tlaxcala, and Hidalgo), North Center (Aguascalientes, Guanajuato, San Luis Potosi, Zacatecas, and Querétaro), South Central (Morelos, State of Mexico, and Mexico City), and South (Guerrero, Oaxaca, Chiapas, Tabasco, Campeche, Quintana Roo, and Yucatán). The last 2 regions (Southwest and Southeast), given their sociodemographic similarities, were included as a single region.

### Ethical Issues

An ethical review board in each country approved the study: in Brazil, INI Evandro Chagas-FIOCRUZ institutional review board (#CAAE 82021918.0.0000.5262); in Mexico, the research ethics committee of the National Institute of Psychiatry *Ramón de la Fuente Muñiz* (#CEI/C/038/2018); and in Peru, Universidad Peruana Cayetano Heredia Ethical Committee for Research with Human Subjects (#101460). All study participants provided their informed consent electronically before initiating the Web-based survey. No identification of participants was collected.

### Statistical Analysis

Participant characteristics, awareness, willingness to use, barriers, and facilitators of PrEP were described by their frequencies, and chi-square tests were used to compare the variables per country (Brazil, Mexico, and Peru). Some survey questions included options such as *I do not want to answer* or *I do not know* to aid participant comfort. These answers were considered missing for data analysis. We compared the main outcome (willingness to use PrEP) by participant characteristics using chi-square tests for each country. Multivariable logistic regression was performed calculating adjusted odds ratios to explore factors associated with willingness to use PrEP for each country. Age, income, and schooling (and race in Brazil) were defined a priori as confounders and were kept in the final model for each country, irrespective of statistical significance. Models were created using a backwards stepwise modeling approach; variables with a bivariate *P* value of less than .01 were included in the initial model and subsequently excluded if their *P* value was greater than .05. Exclusions were done variable by variable, starting by excluding the variable with the highest *P* value and then rerunning the model to repeat the process. The final multivariable models included variables that remained significant (threshold of *P*<.05) after the backwards stepwise process and the a priori defined confounders. All analyses were performed using STATA version 14 (College Station, TX).

## Results

**Figure 1:**
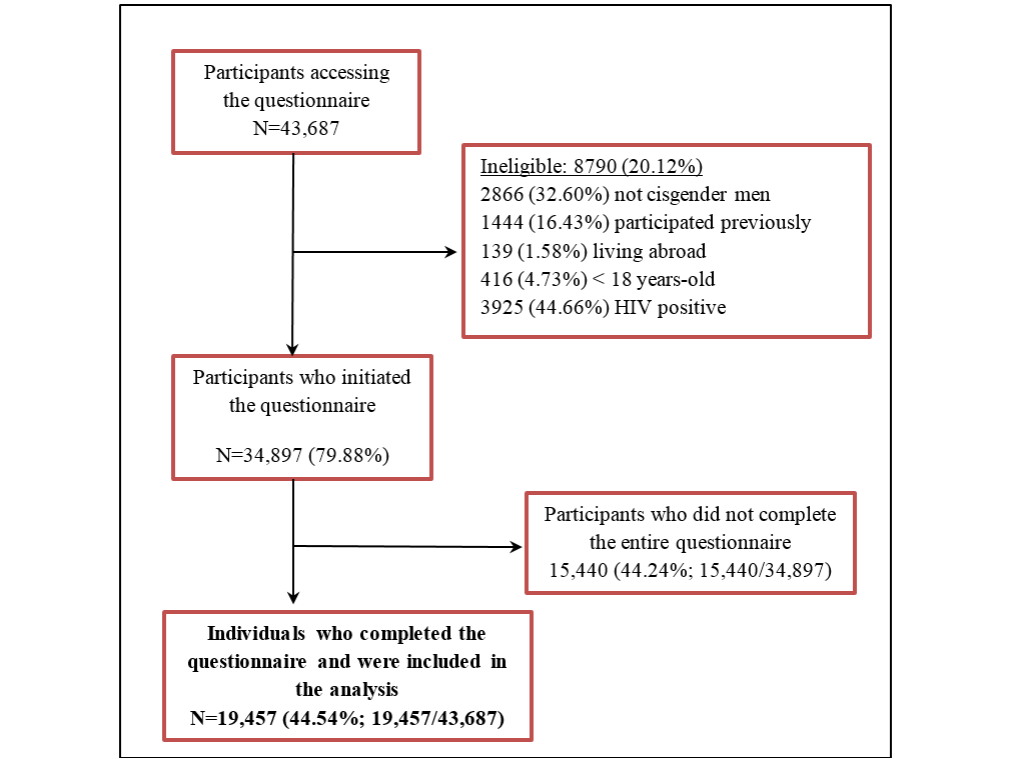
Study flowchart. Brazil, Mexico and Peru, 2018.

During the study, a total of 43,687 individuals provided their informed consent; 20.12% (8790/43,687) were ineligible (Figure 1). Of the 34,897/43,687 (79.88%) eligible individuals, 19,457/43,687 (44.54%) completed the questionnaire and were included in this analysis. Among them, 11,367/19,457 (58.42%) were from Brazil, 5934/19,457 (30.50%) were from Mexico, and 2156/19,457 (11.08%) were from Peru. Almost half of respondents (8981/19,457, 46.17%) lived in the largest urban centers of these countries: São Paulo, Brazil (3198/19,457, 16.44%), Mexico City, Mexico (2618/19,457, 13.46%), Rio de Janeiro, Brazil (1589/19,457, 8.17%), and Lima, Peru (1576/19,457, 8.10%).

Table 1 shows the characteristics of the participants who completed the questionnaire in each country. Their median age was 28 years (interquartile range: 24-34). The proportion of young individuals (18-24 years) was high in Peru (41.23%, 889/2156) and similar in Brazil (28.35%; 3222/11,367) and Mexico (29.76%; 1766/5934). Almost half of respondents from Brazil were white (52.82%, 6004/11,366), whereas the majority from Peru were nonwhite (80.42%, 1672/2079). In Brazil and Peru, fewer individuals were in the high-income category (13.47%, 1531/11,367 and 14.09%; 272/1930, respectively), whereas in Mexico more than a quarter (26.46%, 1419/5363) were in this category. Most participants had at least some postsecondary education in all 3 countries, but this proportion was lower in Brazil (61.14%, 6888/11,266).

Most participants were sexually attracted only to men (89.20%, 17,306/19,401), and the proportion of those attracted to both men and women was highest in Peru (17.13%, 367/2142), followed by Mexico (10.94%, 647/5914) and then Brazil (7.12%, 808/11,348). Among those who were only sexually attracted to women, almost half (45.79%, 125/273) had sex with men in the previous 6 months. Most participants did not have a steady partner (73.57%, 14,195/19,294). Most respondents were recruited on Grindr (68.61%, 13,349/19,457), and this was true for Brazil (67.06%, 7623/11,367) and Mexico (80.91%, 4801/5934), whereas in Peru, the plurality was recruited via Facebook (48.10%, 1037/19,457). Peru has the highest proportion of individuals who never used apps for sexual encounters (19.20%, 414/2156), whereas most Brazilians used them daily (54.05%, 6142/11,363).

Overall, 67.51% (13,110/19,419) of respondents would be eligible for PrEP, whereas only 9.80% (1858/18,959) of participants had high HIV risk perception, which was lowest in Brazil (8.94%, 986/11,029). Almost 20% (3862/19,352) had never tested for HIV, and this was highest in Peru (24.47%, 524/2141). A total of 45.66% (8836/19,352) MSM had tested for HIV in the previous 3 or 6 months, and this was highest in Brazil (48.73%, 5505/11,297). Condomless receptive anal sex was reported by 40.13% (7755/19,326) overall, being higher in Peru (44.48%, 954/2145) than in Mexico (40.04%, 2368/5914) and Brazil (39.34%, 4433/11,268). Even with the relatively high proportion of postexposure prophylaxis (PEP) awareness in Brazil (66.07%, 7510/11,367) and Mexico (55.09%, 3269/5934), ever use of PEP was very low in these countries, with only slightly more than 10% (1452/11,326) in Brazil.

Most MSM had heard about PrEP (64.92%, 12,592/19,396; Table 2); however, this proportion was much lower in Peru 46.60% (1002/2150), compared with over 60% in Brazil (68.82, 7794/11,325) and Mexico (64.11; 3796/5921). The majority had never used PrEP (96.19%, 18,685/19,425). Overall, willingness to use PrEP was reported by 64.23% (12498/19,457); this was highest in Mexico (70.07%, 4158/5934), followed by Brazil (62.45%, 7099/11,367) and Peru (57.56%, 1241/2156). Anticipated risk compensation if taking PrEP was reported by 21.83% (4248/19,457) overall. Injectable PrEP was the preferred PrEP regimen among a plurality of the Brazilians (44.61%, 5071/11,368) and Peruvians (41.00%, 884/2156), whereas daily oral PrEP was preferred among the Mexicans (37.92%, 2260/5960).

All information barriers to PrEP were considered *important* or *very important* by a majority of respondents in each country. There was less importance assigned to the behavioral and belief barriers to PrEP, the barrier *taking pills everyday* was considered *important* or *very important* by 47.90% (9320/19,457) overall, though this was less of a concern in Mexico 42.08% (2497/5934). The majority of respondents in Mexico and Peru were concerned that taking PrEP indicates being at risk of HIV infection and that their partners might expect condomless anal sex. Among the assessed facilitators to PrEP, only 2 were not considered *very important* by the majority of the participants: support for taking daily PrEP from apps or messages and counseling about sex.

**Table 1:** Characteristics of the individuals who completed the questionnaire in Brazil, Mexico, and Peru (2018).

Characteristics			Brazil (N=11,367; 58.42%)	Mexico (N=5,934; 30.50%)	Peru N=2,156; 11.08%)	Total (N=19,457)	P value^a^
**Age (years; n=19,456), median (interquartile range)**			**29 (24-35)**	**28 (24-34)**	**26 (22-31)**	**28 (24-34)**	**N/A^b^**
	18-24		3222 (28.35)	1766 (29.76)	889 (41.23)	5877 (30.21)	<.001
	25-35		5364 (47.19)	2991 (50.40)	970 (44.99)	9325 (47.93)	N/A
	≥36		2780 (24.46)	1177 (19.83)	297 (13.78)	4254 (21.86)	N/A
Race (nonwhite^c^; n=13,446), n (%)			5363 (47.18)	N/A	1672 (80.42)	7035 (52.32)	<.001
**Monthly income^d^** **(n=18,660), n (%)**			**N/A**	**N/A**	**N/A**	**N/A**	**<.001**
	Low		5136 (45.18)	1544 (28.79)	710 (36.79)	7390 (39.60)	—
	Middle		4700 (41.35)	2400 (44.75)	948 (49.12)	7087 (37.98)	N/A
	High		1531 (13.47)	1419 (26.46)	272 (14.09)	4183 (22.42)	N/A
Schooling (≤ secondary education; n=19,300), n (%)			4378 (38.86)	1395 (23.60)	461 (21.69)	6234 (32.30)	<.001
**Sexual attraction (n=19,401), n (%)**			**N/A**	**N/A**	**N/A**	**N/A**	**<.001**
	Men		10,386 (91.54)	5212 (88.14)	1708 (79.74)	17,306 (89.20)	N/A
	Women		152 (1.34)	54 (0.91)	67 (3.13)	273 (1.41)	N/A
	Men/women		808 (7.12)	647 (10.94)	367 (17.13)	1822 (9.39)	N/A
Steady partner (yes; n=19,294), n (%)			2916 (25.83)	1576 (26.77)	607 (28.67)	5099 (26.43)	.02
**Recruitment (n=19,457), n (%)**			**N/A**	**N/A**	**N/A**	**N/A**	**<.001**
	Grindr		7623 (67.06)	4801 (80.91)	925 (42.90)	13,349 (68.61)	N/A
	Hornet		2489 (21.90)	569 (9.59)	20 (0.93)	3078 (15.82)	N/A
	Facebook		780 (6.86)	230 (3.88)	1037 (48.10)	2047 (10.52)	N/A
	Other		475 (4.18)	334 (5.63)	174 (8.07)	983 (5.05)	N/A
**Use of apps for sexual encounters (n=19,454), n (%)**			**N/A**	**N/A**	**N/A**	**N/A**	**<.001**
	Never		835 (7.35)	432 (7.28)	414 (19.20)	1681 (8.64)	N/A
	Sometimes		4387 (38.60)	3336 (56.22)	1033 (47.91)	8756 (45.01)	N/A
	Daily		6142 (54.05)	2166 (36.50)	709 (32.88)	9017 (46.35)	N/A
**Last HIV testing (n=19,352), n (%)**			**N/A**	**N/A**	**N/A**	**N/A**	**<.001**
	Previous 3 months		3516 (31.12)	1428 (21.15)	551 (25.74)	5495 (28.39)	N/A
	Previous 6 months		1989 (17.60)	1031 (17.44)	321 (14.99)	3341 (17.26)	N/A
	Previous 12 months		1835 (16.24)	843 (14.26)	277 (12.94)	2955 (15.27)	N/A
	More than 12 months		1972 (17.45)	1259 (21.29)	468 (21.86)	3699 (19.11)	N/A
	Never		1986 (17.58)	1352 (22.86)	524 (24.47)	3862 (19.96)	N/A
**HIV risk perception^e^** **(n=18,959), n (%)**			**N/A**	**N/A**	**N/A**	**N/A**	**<.001**
	Low		7746 (70.22)	3341 (57.41)	1218 (57.78)	12,305 (64.90)	N/A
	Middle		2299 (20.84)	1845 (31.70)	652 (30.93)	4796 (25.30)	N/A
	High		986 (8.94)	634 (10.89)	238 (11.29)	1858 (9.80)	N/A
Preliminary eligibility for PrEP^f^ (n=19,419), n (%)			7938 (70.03)	3668 (61.85)	1504 (69.82)	13,110 (67.51)	<.001
**Sexual behavior**		
	**Number of male sexual partners^g^** **(n=19,376), n (%)**		**N/A**	**N/A**	**N/A**	**N/A**	**<.001**
		None	873 (7.68)	1401 (23.82)	345 (16.2)	2619 (13.52)	N/A
		1-5	6036 (53.11)	2872 (48.84)	1145 (53.78)	10,053 (51.88)	N/A
		6-10	1958 (17.23)	821 (13.96)	290 (13.62)	3069 (15.84)	N/A
		>10	2499 (21.99)	787 (13.38)	349 (16.39)	3635 (18.76)	N/A
Condomless receptive anal sex^g^ (yes; n=19,326), n (%)			4433 (39.34)	2368 (40.04)	954 (44.48)	7755 (40.13)	<.001
Condomless insertive anal sex^g^ (yes; n=19,322), n (%)			5106 (45.31)	2511 (42.45)	960 (44.90)	8577 (44.39)	<.001
Sex with HIV+ male partner^g^ (yes; n=19,270), n (%)			1217 (10.82)	712 (12.08)	269 (12.64)	2198 (11.41)	.002
Sex with unknown HIV status male partner^g^ (yes; n=19,133), n (%)			3911 (35.27)	2956 (49.98)	1108 (52.02)	7975 (41.68)	<.001
Sex under alcohol use^g^ (yes; n=19,414), n (%)			4190 (36.97)	2089 (35.23)	778 (36.15)	7057 (36.35)	.08
Chemsex^g^ (yes; n=19,401), n (%)			1993 (17.60)	948 (16.00)	243 (11.29)	3184 (16.41)	<.001
Transactional sex^g^ (yes; n=19,456), n (%)			616 (5.42)	271 (4.57)	155 (7.19)	1042 (5.36)	<.001
STI diagnoses^fg,h^ (yes; n=18,875), n (%)			1476 (13.25)	372 (6.52)	197 (9.70)	2045 (10.83)	<.001
PEP^i^ awareness (yes; n=19,457), n (%)			7510 (66.07)	3269 (55.09)	778 (36.09)	11,557 (59.40)	<.001
PEP use (yes; n=19,393), n (%)			1452 (12.82)	310 (5.24)	81 (3.76)	1843 (9.50)	<.001
Binge drinking^g^ (yes; n=19,390), n (%)			7774 (68.39)	4094 (69.43)	1518 (71.40)	13,386 (69.04)	<.001
**Substance use^g^** **(n=18,906), n (%)**			**N/A**	**N/A**	**N/A**	**N/A**	**N/A**
	Tobacco		2905 (25.56)	1981 (35.87)	539 (26.72)	5425 (28.69)	<.001
	Marijuana or hash		2907 (25.57)	1369 (24.79)	471 (23.35)	4747 (25.11)	.09
	Stimulants^j^		1622 (14.27)	545 (9.87)	130 (6.45)	2297 (12.15)	<.001
	Hallucinogens^k^		994 (8.74)	248 (4.49)	28 (1.39)	1270 (6.72)	<.001
	Poppers		735 (6.47)	1233 (22.33)	195 (9.67)	2163 (11.44)	<.001
	Erectile dysfunction drug^g^		1054 (9.27)	605 (10.96)	90 (4.46)	1749 (9.25)	<.001

^a^Chi-square test.

^b^N/A: not applicable.

^c^Black, Asian, Native American, and Mix race; this question was not available for Mexican respondents.

^d^For Brazil, we considered the number of minimum wages in the family monthly income: low ≤3, middle 4-10, high >10 (monthly minimum wage in 2018 was 954 BRL=US $250, currency from June 2018). For Peru, we considered individual monthly income, categorized by number of minimum salaries: low ≤3, middle 4-10, high >10 (monthly minimum wage in 2018 was 850 PEN=US $265). For Mexico, we considered individual monthly income, categorized by number of minimum salaries: low: from no income to <3, middle 3-4, high ≥ 5 (monthly minimum wage in 2018 was 2686 MXN=US $141).

^e^In the next year.

^f^Adapted from the WHO criteria for pre-exposure prophylaxis, which included the following: unprotected anal sex with a male or trans partner, sex with an HIV-positive partner, sex work, or STI diagnosis; all in the past 6 months.

^g^During the previous 6 months.

^h^Syphilis, gonorrhea, or rectal chlamydia.

^i^PEP: postexposure prophylaxis.

^j^Cocaine, crack, ecstasy, and GHB (4-hydroxybutanoic acid).

^k^Solvents, lysergic acid diethylamide, and ketamine.

**Table 2:** Awareness, willingness to use, barriers, and facilitators to daily oral pre-exposure prophylaxis in Brazil, Mexico, and Peru (2018).

Variables			Brazil (N=11,367; 58.42%), n (%)	Mexico (N=5934; 30.50%), n (%)	Peru (N=2156; 11.08%), n (%)	Total (N=19,457), n (%)	*P* value^a^
PrEP^b^ awareness (yes; n=19,396^c^)			7794 (68.82)	3796 (64.11)	1002 (46.60)	12,592 (64.92)	<.001
Willingness to use PrEP (yes)			7099 (62.45)	4158 (70.07)	1241 (57.56)	12,498 (64.23)	<.001
**PrEP use (n=19,425)**							**<.001**
	Never		10,875 (95.67)	5735 (96.97)	2075 (96.78)	18,685 (96.19)	N/A^d^
	Current		266 (2.34)	87 (1.47)	42 (1.96)	395 (2.03)	N/A
	Past		226 (1.99)	92 (1.56)	27 (1.26)	345 (1.78)	N/A
Anticipated risk compensation (yes)			2325 (20.45)	1380 (23.26)	543 (25.19)	4248 (21.83)	<.001
PrEP if part of Ministry of Health program (yes)			5814 (51.15)	4113 (69.31)	1165 (54.04)	11,092 (57.01)	<.001
**Preferred PrEP regimen**							**<.001**
	Daily oral		3802 (33.45)	2260 (37.92)	719 (33.35)	6771 (34.80)	N/A
	Event-driven		2494 (21.94)	1491 (25.13)	553 (25.65)	4538 (23.32)	N/A
	Injectable		5071 (44.61)	2193 (36.96)	884 (41.00)	8148 (41.88)	N/A
**Barriers to daily oral PrEP^e^**							**N/A**
	**Information**						
		Afraid of short-term side effects	6805 (59.87)	4126 (69.53)	1614 (74.86)	12,545 (64.48)	<.001
		Afraid of long-term side effects	7601 (66.87)	4601 (77.54)	1721 (79.82)	13,923 (71.56)	<.001
		Afraid that antiretroviral therapy would not work if infected	6522 (57.38)	5031 (84.78)	1810 (83.95)	13,363 (68.68)	<.001
		Afraid of not being 100% protected against HIV	7831 (68.89)	4900 (82.57)	1794 (83.21)	14,525 (74.65)	<.001
	**Behaviors**						
		Taking pills everyday	5774 (50.80)	2497 (42.08)	1049 (48.65)	9320 (47.90)	<.001
		Talking to a doctor about sex life	2670 (23.49)	1897 (31.97)	886 (41.09)	5453 (28.03)	<.001
		Having HIV/ sexually transmitted infection testing every 3 months	5025 (44.21)	2615 (44.07)	1120 (51.95)	8760 (45.02)	<.001
	**Beliefs**						
		Taking PrEP means that I am at risk of HIV infection	4540 (39.94)	3339 (56.27)	1203 (55.80)	9082 (46.68)	<.001
		My partners may expect condomless anal sex	4310 (37.92)	3584 (60.40)	1190 (55.19)	9084 (46.69)	<.001
		Afraid that people may think I am HIV+	4303 (37.86)	1966 (33.13)	950 (44.06)	7219 (37.10)	<.001
		Afraid that people ask me why I am using PrEP when they see me taking the pills	3709 (32.63)	1663 (28.02)	880 (40.82)	6252 (32.13)	<.001
**Facilitators to daily oral PrEP^f^**							**N/A**
	Free PrEP		8797 (77.39)	4317 (72.75)	1474 (68.37)	14,588 (74.98)	<.001
	Access to free HIV test		8573 (75.42)	4554 (76.74)	1567 (72.68)	14,694 (75.52)	.001
	Access to other free exams		8052 (70.84)	4285 (72.21)	1499 (69.53)	13,836 (71.11)	.04
	Access to personal PrEP counseling		7103 (62.49)	4129 (69.58)	1444 (66.98)	12,676 (65.15)	<.001
	Support from apps or messages		5464 (48.07)	2880 (48.53)	1160 (53.80)	9504 (48.85)	<.001
	Support and counseling about my sex life		4272 (37.58)	3138 (52.88)	1175 (54.50)	8585 (44.12)	<.001

^a^Chi-square test.

^b^PrEP: pre-exposure prophylaxis.

^c^The full sample, N=19,457, is included unless otherwise specified.

^d^N/A: not applicable.

^e^Barriers were grouped into PrEP information, beliefs, and behaviors. The percent shown is of participants responding that the barrier was *important* or *very important*.

^f^The percent shown is of participants responding that the facilitator was *very important*.

**Figure 2:**
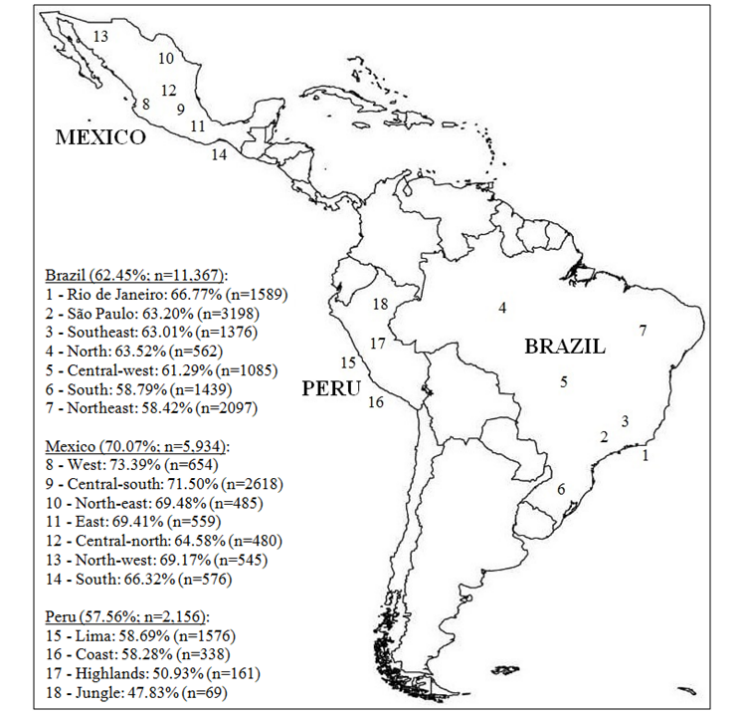
Willingness to use daily oral pre-exposure prophylaxis per region. Brazil, Mexico and Peru, 2018. Percentage of willingness to use daily oral prep is given per region; n is total number of participants per region or country.

The proportion of individuals willing to use PrEP by characteristics and country are provided in the Multimedia Appendix 1. There were geographic differences in willingness to use PrEP (see Figure 2). In Brazil, willingness to use PrEP varied across the country, being higher in Rio de Janeiro (66.77%, 1061/1589) and São Paulo (63.20%, 2021/3198; *P*<.001). In Mexico, it was higher in the West (73.39%, 480/654) and Central-South (71.50%, 1872/2618), but lower in Central-North (64.58%, 310/480; *P*=.002). There was no statistically significant difference by region in Peru (*P*=.09).

In each country’s final multivariable model, PrEP awareness and PrEP facilitators were positively associated, whereas behavioral and belief barriers were negatively associated with willingness to use PrEP. HIV risk perception was positively associated with willingness to use PrEP in Brazil and Peru, whereas daily use of apps for sexual encounters was positively associated only in Mexico and Peru. HIV testing at least once in a lifetime was positively associated with willingness to use PrEP only in Brazil. Mexico was the only country where age was significantly associated with willingness to use PrEP, with the older age categories (25+ years) reporting significantly less willingness compared with the youngest category (18-24 years). Informational barriers were negatively associated in Brazil but positively associated with willingness to use PrEP in Peru (Table 3).

**Table 3:** Factors associated with willingness to use daily oral pre-exposure prophylaxis in Brazil, Mexico, and Peru (2018).

Variable Variable		Brazil		Mexico		Peru	
		Bivariate models, OR^a^ (95% CI)	Multivariate model, AOR^b^ (95% CI)^c^	Bivariate models, OR (95% CI)	Multivariate model, AOR (95% CI)^c^	Bivariate models, OR (95% CI)	Multivariate model, AOR (95% CI)^c^
**Age (years)**							
	18-24	Ref.^d^	Ref.	Ref.	Ref.	Ref.	Ref.
	25-35	*1.11 (1.02-1.21)*	0.95 (0.86-1.06)	1.06 (0.93-1.21)	*0.84 (0.71-0.98)*	*1.32 (1.10-1.59)*	1.06 (0.85-1.34)
	≥36	*1.13 (1.01-1.25)*	0.97 (0.86-1.10)	0.95 (0.81-1.11)	*0.81 (0.66-0.99)*	*1.47 (1.13-1.93)*	1.36 (0.97-1.90)
Color (nonwhite^e^ vs white)		0.95 (0.88-1.02)	1.01 (0.93-1.10)	N/A^f^	N/A	0.88 (0.70-1.09)	—^g^
**Monthly income^h^**							
	Low	Ref.	Ref.	Ref.	Ref.	Ref.	Ref.
	Middle	*1.20 (1.11-1.31)*	1.08 (0.99-1.19)	1.05 (0.90-1.23)	0.99 (0.84-1.18)	*1.37 (1.12-1.66)*	*1.28 (1.02-1.63)*
	High	*1.31 (1.16-1.47)*	*1.27 (1.10-1.46)*	1.11 (0.96-1.28)	0.92 (0.78-1.11)	*1.51 (1.14-2.01)*	1.20 (0.84-1.71)
Schooling (any postsecondary education vs ≤ secondary education)		*1.15 (1.07-1.25)*	0.99 (0.90-1.08)	*1.24 (1.09-1.41)*	1.07 (0.92-1.26)	1.19 (0.96-1.46)	0.86 (0.66-1.12)
Steady partner (yes vs no)		1.06 (0.97-1.16)	—	*1.14 (1.01-1.30)*	—	1.05 (0.87-1.27)	—
HIV testing (at least once lifetime vs never)		*1.48 (1.34-1.63)*	*1.12 (1.00-1.25)*	*1.42 (1.25-1.61)*	—	*1.36 (1.12-1.66)*	—
**Use of apps for sexual encounters**							
	Never	Ref.	—	Ref.	Ref.	Ref.	Ref.
	Sometimes	1.06 (0.91-1.23)	—	*1.38 (1.13-1.70)*	1.15 (0.90-1.47)	*1.32 (1.05-1.66)*	1.18 (0.91-1.53)
	Daily	*1.39 (1.20-1.62)*	—	*1.84 (1.48-2.28)*	*1.34 (1.04-1.73)*	*1.65 (1.29-2.11)*	*1.34 (1.01-1.78)*
**HIV risk perception^i^**							
	Low	Ref.	Ref.	Ref.	—	Ref.	Ref.
	Middle	*1.38 (1.26-1.53)*	*1.18 (1.06-1.31)*	*1.34 (1.18-1.51)*	—	*1.34 (1.11-1.63)*	*1.32 (1.06-1.63)*
	High	*1.99 (1.71-2.31)*	*1.53 (1.30-1.80)*	*1.75 (1.43-2.14)*	—	*1.83 (1.36-2.46)*	*1.58 (1.14-2.19)*
Preliminary eligibility for PrEP^j^ (yes vs no)		*1.62 (1.50-1.76)*	*1.33 (1.22-1.46)*	*1.47 (1.32-1.65)*	*1.29 (1.13-1.47)*	*1.20 (1.00-1.44)*	—
Number of male sexual partners^k^ (>5 vs ≤5)		*1.55 (1.44-1.68)*	*1.19 (1.09-1.30)*	*1.60 (1.40-1.83)*	*1.18 (1.01-1.38)*	*1.42 (1.17-1.71)*	—
Sex under alcohol use^k^ (yes vs no)		*1.16 (1.07-1.26)*	—	*1.17 (1.03-1.33)*	—	1.08 (0.89-1.30)	—
Chemsex^k^ (yes vs no)		*1.24 (1.12-1.38)*	—	*1.48 (1.25-1.76)*	—	*1.44 (1.09-1.92)*	—
PrEP awareness (yes vs no)		*1.98 (1.82-2.14)*	*1.66 (1.52-1.81)*	*2.37 (2.11-2.66)*	*2.05 (1.79-2.34)*	*2.03 (1.70-2.42)*	*1.84 (1.51-2.26)*
Anticipated risk compensation (yes vs no)		*1.48 (1.34-1.63)*	*1.32 (1.18-1.47)*	*1.37 (1.19-1.57)*	*1.37 (1.17-1.59)*	*1.24 (1.02-1.52)*	—
Barriers: Information, mean (SD)		*0.94 (0.93-0.95)*	*0.95 (0.94-0.97)*	*0.97 (0.96-0.99)*	—	*1.03 (1.01-1.06)*	*1.05 (1.01-1.08)*
Barriers: Behaviors, mean (SD)		*0.93 (0.92-0.94)*	*0.95 (0.94-0.96)*	*0.92 (0.90-0.93)*	*0.94 (0.92-0.95)*	*0.95 (0.93-0.97)*	*0.95 (0.92-0.98)*
Barriers: Believes, mean (SD)		*0.95 (0.94-0.95)*	*0.97 (0.96-0.98)*	*0.93 (0.91-0.94)*	*0.95 (0.93-0.97)*	*0.95 (0.93-0.97)*	*0.95 (0.92-0.97)*
Facilitators, mean (SD)		*1.07 (1.06-1.08)*	*1.09 (1.08-1.10)*	*1.07 (1.05-1.08)*	*1.11 (1.09-1.12)*	*1.07 (1.05-1.09)*	*1.11 (1.08-1.14)*

^a^OR: odds ratio.

^b^AOR: adjusted odds ratio.

^c^Variables with *P*<.01 in bivariate models were included in the initial multivariable model. Variables with *P*<.05 were kept in the final multivariable models, excepted for age, monthly income, and schooling defined a priori for all countries, and race only for Brazil; statistically significant associations at *P*<.05 in italics. Region did not remain in the final multivariate models and bivariate analysis is not shown.

^d^Ref.: reference.

^e^Black, Asian, Native American, or Mix race.

^f^N/A: not applicable.

^g^Not statistically significant.

^h^For Brazil, we considered the number of minimum wages in the family monthly income: low ≤3, middle 4-10, high >10 (monthly minimum wage in 2018 was 954 BRL=US $250, currency from June 2018). For Peru, we considered individual monthly income, categorized by number of minimum salaries: low ≤3, middle 4-10, high >10 (monthly minimum wage in 2018 was 850 PEN=US $265). For Mexico, we considered individual monthly income, categorized by number of minimum salaries: low, from no income to <3, middle 3-4, high ≥ 5 (monthly minimum wage in 2018 was 2686 MXN=US $141).

^i^In the next 12 months.

^j^Adapted from the WHO criteria for pre-exposure prophylaxis, which included the following: unprotected anal sex with a male or trans partner, sex with an HIV-positive partner, sex work, or STI diagnosis; all in the past 6 months.

^k^During the previous 6 months.

## Discussion

This study shows that MSM from Brazil, Mexico, and Peru are willing to use daily oral PrEP. Willingness to use PrEP was higher in Mexico, followed by Brazil and then Peru. There were similarities in the factors associated with willingness to use PrEP across the 3 countries, and the most important factor was PrEP awareness. It is likely that as PrEP awareness increases in these settings, willingness to use PrEP will also increase. This trend was observed in Brazil [20,21] and in a recent meta-analysis evaluating the factors associated with willingness to use PrEP in low- and middle-income settings [17].

In our models, younger age was significantly associated with willingness to use PrEP in Mexico but not in Brazil and Peru. This is a concern as younger MSM are at high risk of HIV acquisition in Latin America [28] and are experiencing an increase in HIV cases in Brazil [7], Mexico [29], and Peru [30]. The association of income with willingness was different by country; higher income was associated with willingness in Peru and Brazil, whereas there was no association in Mexico. To improve equity in PrEP access, interventions to increase knowledge of PrEP among lower-income and young MSM are essential, and this could be achieved with community-based educators and Web-based advertisements, as internet access is becoming more available in Latin America [31]. Nevertheless, this finding should be interpreted with caution as respondents with lower income may associate their willingness to use PrEP with accessibility to PrEP free of charge within the public health system. Of note, more Mexican MSM reported interest in PrEP as part of a MoH program (free of charge) than Brazilians or Peruvians. In addition, almost 75% of the entire sample thought that PrEP at no cost is *very important* or *important*.

Our regression models captured that informational barriers were significantly associated with willingness to use PrEP in Peru. This unexpected result is likely owing to the limited available information, reflected by the low observed PrEP awareness. In all 3 countries, higher HIV risk perception and higher risky sexual behavior were associated with willingness to use PrEP, reflecting that the individuals to whom PrEP is targeted are those willing to use it. This is corroborated by other studies accessing willingness to use PrEP [21,22,32]. Anticipated risk compensation (the possibility of not using condoms while on PrEP) was associated with willingness in Brazil and Mexico but not in Peru. Risk compensation is a recurrent concern related to PrEP, and evidence of this was recently observed in other studies [33,34]; conversely, there was no statistically significant increase in condomless receptive anal sex during the PrEP Brazil study [35] or in a study conducted in Thailand [36]. Risk compensation continues to require monitoring and adequate PrEP education.

Notably in Brazil and Peru, the majority of participants would prefer injectable PrEP, and for Mexico, it was the second preferred regimen, just slightly below daily oral PrEP. Event-driven PrEP was third in all 3 countries. Injectable PrEP regimens, currently under study, may be useful and acceptable for MSM who have shown low adherence for oral PrEP regimens. As new PrEP technologies become available, it will be important to continue providing education and collecting information on preferences.

The samples included from each country were distinct; most likely, this was related to the recruitment methods. The fact that a higher proportion of lower-income MSM was included in Brazil could be explained by the increase of internet access in this country, where it is estimated 1 smartphone per person [31]. As expected, most of respondents are from each country’s urban large regions where more MSM are concentrated, as well as where access to internet is easier. Brazilian MSM reported more male sexual partners than Mexican and Peruvians but lower rates of steady partner and condomless receptive anal sex. In a recent, large sample of MSM in Brazil, having more sex partners was associated with condomless receptive anal sex among MSM aged ≥25 years but not among those aged 18 to 24 years [37]. The higher proportions of STI diagnoses and PEP use in Brazil could be explained by the higher availability and access to exams and PEP free of charge in this country, especially in the Southeast region where most respondents live. Peruvian MSM reported more condomless receptive anal sex, transactional sex, binge drinking, and more had never tested for HIV than in the other countries.

Some study limitations should be considered. First, Web-based studies are not probabilistic sampling strategies, precluding the generalization of the findings. Given the cross-sectional nature of the data, causality and the direction of association may not be inferred. All collected data were self-reported by participants and may be subject to bias. Our data are subject to recall bias owing to 6-month or 12-month recall periods. There is also a concern about participants taking the survey multiple times. To mitigate this bias, the first question of the survey was as follows: *Are you answering this survey for the first time?* (3.3% of participants answered *no* and were excluded from the study). Finally, we have no ability to assess the veracity of respondents truly being MSM; the analysis included self-identified MSM independent of their sexually activity in the previous 6 months or their self-identified sexual attraction. There were respondents who reported being sexually attracted only to women–though importantly, among this group, 45.8% (125/273) reported having sex with a man in the past 6 months. We believe the population of interest were reached by and responded to this survey.

### Conclusions

In this first cross-country, Web-based survey in Latin America willingness to use PrEP was found to be high and directly related to levels of awareness. Interventions to increase awareness and PrEP knowledge about safety and efficacy are crucial to increase PrEP demand among MSM at high risk who can benefit from this prevention technology. Social media campaigns and campaigns in STI clinics may be valuable to achieve this goal. This study presents important information to support the implementation of PrEP for MSM in Brazil, Mexico, and Peru.

## Appendix

Multimedia Appendix 1 Proportion of individuals willing to use pre-exposure prophylaxis by characteristics and country.

## Abbreviations

FTC/TDF: emtricitabine/tenofovir
ImPrEP: implementation PrEP
MoH: Ministry of Health
MSM: men who have sex with men
PEP: postexposure prophylaxis
PrEP: pre-exposure prophylaxis
STI: sexually transmitted infection
WHO: World Health Organization
