# Authorship Correction: Factors Associated With Willingness to Use Pre-Exposure Prophylaxis in Brazil, Mexico, and Peru: Web-Based Survey Among Men Who Have Sex With Men

**DOI:** 10.2196/15504

**Published:** 2019-07-18

**Authors:** Thiago Silva Torres, Kelika A Konda, E Hamid Vega-Ramirez, Oliver A Elorreaga, Dulce Diaz-Sosa, Brenda Hoagland, Steven Diaz, Cristina Pimenta, Marcos Benedetti, Hugo Lopez-Gatell, Rebeca Robles-Garcia, Beatriz Grinsztejn, Carlos Caceres, Valdilea G Veloso, ImPrEP Study Group

**Affiliations:** 1 Instituto Nacional de Infectologia Evandro Chagas, Fundação Oswaldo Cruz (INI/Fiocruz) Rio de Janeiro Brazil; 2 Centro de Investigación Interdisciplinaria en Sexualidad Sida y Sociedad, UPCH Lima Peru; 3 Condesa & Condesa-Iztapalapa Specialized Clinics Mexico City Mexico; 4 National Institute of Psychiatry Ramon de la Fuente Muñiz Mexico City Mexico; 5 Center for Prevention and Comprehensive Healthcare for HIV/AIDS of Mexico City Mexico City Mexico; 6 Brazilian Ministry of Health Brasília Brazil; 7 National Institute of Public Health Mexico City Mexico

In “Factors Associated With Willingness to Use Pre-Exposure Prophylaxis in Brazil, Mexico, and Peru: Web-Based Survey Among Men Who Have Sex With Men” (JMIR Public Health Surveill 2019;5(2):e13771), the metadata information for author Cristina Pimenta (listed in 8th position) was accidentally overwritten by a duplicate of author Marcos Benedetti (listed in 9th position) when attempting to correct the spelling of Marcos’ surname.

Authorship was previously as follows:

Thiago Silva Torres, Kelika A Konda, E Hamid Vega-Ramirez, Oliver A Elorreaga, Dulce Diaz-Sosa, Brenda Hoagland, Steven Diaz, Marcos Benedetti, Marcos Bennedeti, Hugo Lopez-Gatell, Rebeca Robles-Garcia, Beatriz Grinsztejn, Carlos Caceres, Valdilea G Veloso, ImPrEP Study Group

The duplicate name and associated information for the author in 8th position has been adjusted to list Cristina Pimenta, PhD, with affiliation “Brazilian Ministry of Health, Brasília, Brazil”, and the spelling of “Bennedeti” for the author in 9th position has been corrected to “Benedetti”. Updated authorship is now as follows:

Thiago Silva Torres, Kelika A Konda, E Hamid Vega-Ramirez, Oliver A Elorreaga, Dulce Diaz-Sosa, Brenda Hoagland, Steven Diaz, Cristina Pimenta, Marcos Benedetti, Hugo Lopez-Gatell, Rebeca Robles-Garcia, Beatriz Grinsztejn, Carlos Caceres, Valdilea G Veloso, ImPrEP Study Group

The correction will appear in the online version of the paper on the JMIR website on July 18, 2019, together with the publication of this correction notice. Because this was made after submission to PubMed, PubMed Central, and other full-text repositories, the corrected article also has been resubmitted to those repositories.

