# Supplementary material for: Factors Associated With Willingness to Use Pre-Exposure Prophylaxis in Brazil, Mexico, and Peru: Web-Based Survey Among Men Who Have Sex With Men
Source: JMIR Public Health Surveill. 2019 Jun 17;5(2):e13771. doi: 10.2196/13771 (PMC6601256; doi:10.2196/13771)
Supplement: Multimedia Appendix 1 [file publichealth_v5i2e13771_app1.docx]

**Supplementary material. Proportion of participants willing to use daily oral PrEP by characteristics for Brazil, Mexico and Peru, 2018.**

| Variable | **Brazil** | | | **Mexico** | | | **Peru** | | |
| --- | --- | --- | --- | --- | --- | --- | --- | --- | --- |
| Willingness to use PrEP | No  n=4268 (37.6%) | Yes  n=7099 (62.5%) | p-value | No  n=1776 (29.9%) | Yes  n=4158 (70.1%) | p-value | No  n=915 (42.4%) | Yes  n=1241 (57.6%) | p-value |
| Age (years) |  |  | 0.03 |  |  | 0.30 |  |  | 0.01 |
| 18-24 | 1270 (39.4) | 1952 (60.6) |  | 536 (30.4) | 1,230 (69.7) |  | 416 (46.8) | 473 (53.2) |  |
| 25-35 | 1980 (37.0) | 3384 (63.1) |  | 870 (29.1) | 2121 (70.9) |  | 388 (40.0) | 582 (60.0) |  |
| ≥ 36 | 1018 (37.6) | 1762 (63.5) |  | 370 (31.4) | 807 (68.6) |  | 111 (37.4) | 186 (62.6) |  |
| Race |  |  | 0.17 |  |  |  |  |  | 0.24 |
| White | 2219 (37.0) | 3785 (63.0) |  | NA | NA | NA | 162 (39.8) | 245 (60.2) |  |
| Non-white | 2049 (38.2) | 3314 (61.8) |  | NA | NA | NA | 719 (43.0) | 953 (57.0) |  |
| Monthly income ^c^ |  |  | <0.001 |  |  | 0.35 |  |  | 0.01 |
| Low | 2065 (40.2) | 3071 (59.8) |  | 468 (30.3) | 1076 (69.7) |  | 335 (47.2) | 375 (52.8) |  |
| Middle | 1683 (35.8) | 3017 (64.2) |  | 695 (29.0) | 1705 (71.0) |  | 375 (39.6) | 573 (60.5) |  |
| High | 520 (37.6) | 1011 (66.0) |  | 396 (27.9) | 1023 (72.1) |  | 101 (37.1) | 171 (62.9) |  |
| Schooling |  |  | <0.001 |  |  | 0.01 |  |  | 0.11 |
| ≤ secondary education | 1728 (39.5) | 2650 (60.5) |  | 466 (33.4) | 929 (66.6) |  | 211 (45.8) | 250 (54.2) |  |
| any post-secondary education | 2489 (36.1) | 4398 (63.9) |  | 1310 (28.9) | 3229 (71.1) |  | 692 (41.6) | 972 (58.4) |  |
| Steady partner |  |  | 0.17 |  |  | 0.04 |  |  | 0.59 |
| Yes | 1062 (25.1) | 1854 (26.3) |  | 439 (27.9) | 1137 (72.1) |  | 252 (41.5) | 355 (58.5) |  |
| No | 3170 (37.9) | 5204 (62.1) |  | 1320 (30.6) | 2991 (69.4) |  | 646 (42.8) | 864 (57.2) |  |
| HIV testing |  |  | <0.001 |  |  | <0.001 |  |  | 0.01 |
| Never | 898 (45.2) | 1088 (54.8) |  | 477 (35.6) | 863 (64.4) |  | 252 (48.1) | 272 (51.9) |  |
| Yes | 3336 (35.8) | 5976 (64.2) |  | 1273 (28.0) | 3263 (71.9) |  | 654 (40.5) | 963 (59.6) |  |
| Use of apps for sexual encounters |  |  | <0.001 |  |  | <0.001 |  |  | <0.001 |
| Never | 353 (42.3) | 482 (57.7) |  | 168 (39.0) | 264 (61,1) |  | 206 (49.8) | 208 (50.2) |  |
| Sometimes | 1797 (41.0) | 2590 (59.0) |  | 1051 (31.5) | 2285 (68.5) |  | 443 (42.9) | 590 (57.1) |  |
| Daily | 2115 (34.4) | 4027 (65.6) |  | 557 (25.7) | 1609 (74.2) |  | 266 (37.5) | 443 (62.5) |  |
| HIV risk perception ^d^ |  |  | <0.001 |  |  | <0.001 |  |  | <0.001 |
| Low | 3135 (40.5) | 4611 (59.5) |  | 1098 (32.9) | 2243 (67.1) |  | 562 (46.1) | 656 (53.9) |  |
| Middle | 755 (32.8) | 1544 (67.2) |  | 497 (26.9) | 1348(73.1) |  | 254 (39.0) | 398 (61.0) |  |
| High | 251 (25.5) | 735 (74.5) |  | 139 (21.9) | 495 (78.1) |  | 76 (31.9) | 162 (68.1) |  |
| Preliminary eligibility for PrEP ^e^ |  |  | <0.001 |  |  | <0.001 |  |  | 0.06 |
| No | 1549 (45.6) | 1848 (54.4) |  | 792 (35.0) | 1470 (65.0) |  | 296 (45.5) | 354 (54.5) |  |
| Yes | 2702 (34.0) | 5236 (66.0) |  | 982 (26.8) | 2686 (73.2) |  | 618 (41.1) | 886 (58.9) |  |
| Number of male sexual partners ^h^ |  |  | <0.001 |  |  | <0.001 |  |  | <0.001 |
| <5 | 2869 (41.5) | 4040 (58.5) |  | 1393 (32.6) | 2880 (67.4) |  | 668 (44.8) | 822 (55.2) |  |
| >5 | 1398 (31.4) | 3059 (68.6) |  | 373 (23.2) | 1235 (76.8) |  | 233 (36.5) | 406 (63.5) |  |
| Sex under alcohol use ^f^ |  |  | <0.001 |  |  | 0.02 |  |  | 0.43 |
| Yes | 1441 (37.8) | 2749 (41.4) |  | 553 (26.5) | 1536 (73.5) |  | 316 (42.0%) | 462 (43.8%) |  |
| No | 2374 (37.9) | 3895 (62.1) |  | 723 (29.6) | 1717 (70.4) |  | 437 (42.5) | 592 (57.5) |  |
| Chemsex ^f^ |  |  | <0.001 |  |  | <0.001 |  |  | 0.011 |
| Yes | 648 (32.5) | 1345 (67.5) |  | 211 (22.3) | 737 (77.7) |  | 83 (34.2) | 160 (65.8) |  |
| No | 3163 (37.4) | 5295 (62.6) |  | 1066 (29.8) | 2510 (70.2) |  | 670 (42.8) | 894 (57.2) |  |
| Transactional sex ^f^ |  |  | 0.10 |  |  | 0.04 |  |  | 0.56 |
| Yes | 206 (33.4) | 410 (66.6) |  | 62 (22.9) | 209 (77.1) |  | 68 (43.9) | 87 (56.1) |  |
| No | 3628 (36.7) | 6249 (63.3) |  | 1216 (28.5) | 3046 (71.5) |  | 686 (41.4) | 970 (58.6) |  |
| STI diagnosis ^f, g^ |  |  | <0.001 |  |  | 0.91 |  |  | 0.52 |
| Yes | 425 (28.8) | 1051 (71.2) |  | 110 (29.6) | 262 (70.4) |  | 79 (40.10) | 118 (59.90) |  |
| No | 3747 (38.8) | 5920 (61.2) |  | 1591 (29.9) | 3739 (70.2) |  | 779 (42.5) | 1054 (57.5) |  |
| PrEP awareness |  |  | <0.001 |  |  | <0.001 |  |  | <0.001 |
| Yes | 2526 (32.4) | 5268 (67.6) |  | 881 (23.2) | 2915 (76.8) |  | 578 (50.4) | 570 (49.7) |  |
| No | 1718 (48.7) | 1813 (51.4) |  | 887 (41.7) | 1238 (58.3) |  | 334 (33.3) | 668 (66.7) |  |
| Anticipated Risk Compensation |  |  | <0.001 |  |  | <0.001 |  |  | 0.031 |
| Yes | 709 (30.5) | 1616 (69.5) |  | 346 (25.1) | 1034 (74.9) |  | 209 (38.5) | 334 (61.5) |  |
| No | 3559 (39.4) | 5483 (60.6) |  | 1430 (31.4) | 3124 (68.6) |  | 706 (43.8) | 907 (56.2) |  |
| Barriers |  |  |  |  |  |  |  |  |  |
| Information (Mean, SD) | 15.5 (3.5) | 14.6 (4.0) | <0.001 | 16.9 (3.0) | 16.7 (3.3) | 0.01 | 16.8 (3.3) | 17.1 (3.2) | 0.01 |
| Behaviors (Mean, SD) | 9.1 (2.9) | 8.4 (3.4) | <0.001 | 9.4 (3.5) | 8.1 (4.1) | <0.001 | 9.9 (3.3) | 9.1 (3.7) | <0.001 |
| Believes (Mean, SD) | 11.9 (4.1) | 10.9 (4.5) | <0.001 | 13.1 (4.2) | 11.7 (4,3) | <0.001 | 13.4 (4.1) | 12.4 (4.5) | <0.001 |
| Facilitators (Mean, SD) | 25.1 (4.8) | 26.4 (4.2) | <0.001 | 25.9 (4.2) | 27.0 (3.78) | <0.001 | 25.9 (4.5) | 27.1 (3.9) | <0.001 |

^a^Southeast Brazil, Asian, Native American and Mix race; ^c^For Brazil, we considered the number of minimum wages in the family monthly income: low ≤ 3; middle 4-10; high > 10 (minimum wage in 2018 was 954 BRL=250 USD, currency from June 2018). For Peru, we considered individual monthly income, categorized by number of minimum salaries: low ≤ 3; middle 4-10; high > 10 (minimum wage in 2018 was 850 PEN=265 USD). For Mexico, we considered individual monthly income, categorized by number of minimum salaries: low, from no income to < 3; middle 3-4; high ≥ 5 (minimum wage in 2018 was 2686 MXN = 141 USD); ^d^ In the next months; ^e^ ImPrEP inclusion criteria included: unprotected anal sex with a male or trans partner, sex with an HIV positive partner, sex work, or STI diagnosis; all in the past 6 months. ^f^ During the previous 6 months; ^g^ syphilis, gonorrhea or rectal chlamydia.
